# Signal-to-background ratio and lateral resolution in deep tissue imaging by optical coherence microscopy in the 1700 nm spectral band

**DOI:** 10.1038/s41598-019-52175-9

**Published:** 2019-11-05

**Authors:** Masahito Yamanaka, Naoki Hayakawa, Norihiko Nishizawa

**Affiliations:** Department of Electronics, Nagoya University, Furo-cho, Chikusa-ku, Nagoya, Aichi 464-8603 Japan

**Keywords:** Optical imaging, Confocal microscopy, Imaging and sensing

## Abstract

We quantitatively investigated the image quality in deep tissue imaging with optical coherence microscopy (OCM) in the 1700 nm spectral band, in terms of the signal-to-background ratio (SBR) and lateral resolution. In this work, to demonstrate the benefits of using the 1700 nm spectral band for OCM imaging of brain samples, we compared the imaging quality of OCM *en-face* images obtained at the same position by using a hybrid 1300 nm/1700 nm spectral domain (SD) OCM system with shared sample and reference arms. By observing a reflective resolution test target through a 1.5 mm-thick tissue phantom, which had a similar scattering coefficient to brain cortex tissue, we confirmed that 1700 nm OCM achieved an SBR about 6-times higher than 1300 nm OCM, although the lateral resolution of the both OCMs was similarly degraded with the increase of the imaging depth. Finally, we also demonstrated high-contrast deep tissue imaging of a mouse brain at a depth up to 1.8 mm by using high-resolution 1700 nm SD-OCM.

## Introduction

Optical coherence microscopy (OCM) is a label-free high-resolution imaging modality based on optical coherence tomography (OCT) and confocal microscopy^1,2^. In OCM, the combination of coherence and confocal gating with a high numerical aperture (NA) objective lens enables us to achieve high spatial resolution and image contrast. Currently, OCM imaging techniques are utilized in a wide variety of studies in life science, which requires the visualization of structures with high spatial resolution in a label-free manner.

To visualize structures in deep parts of turbid scattering tissue, the 1300 nm spectral band is commonly used due to the low scattering coefficient compared with that of shorter near-infrared spectral bands, such as 800–1000 nm, which is one of the standard spectral bands used for OCT and OCM imaging^3^. For example, 1300 nm OCM realized both high resolution and large penetration depth (~1.3 mm) in imaging of a rat brain^4^. In OCT studies, a penetration depth of 2.3 mm in mouse brain imaging was demonstrated by using a swept laser source in the 1300 nm spectral band^5^. Despite the relatively higher water absorption coefficient, recently, it has been reported that the use of the 1700 nm spectral band offers a large penetration depth in imaging of turbid scattering tissue^6–9^. In an investigation of 1700 nm swept source OCT, it has also been reported that image contrast is improved compared with 1300 nm SS-OCT, and in addition, a penetration depth of 2.6 mm in mouse brain vascular imaging has also been demonstrated by using optical coherence Doppler tomography^10^. In our group, we have been developing high-resolution 1700 nm OCT by using a supercontinuum (SC) fiber laser source, and we realized a large penetration depth with an axial resolution of 3–5 µm in tissue in which the refractive index was assumed to be 1.38^11–17^. By using an SC source in the 1700 nm spectral band, we also developed 1700 nm time domain (TD) OCM with 1.3 µm lateral and 2.8 µm axial resolutions in tissue. Our OCM study also showed that 1700 nm TD-OCM can realize a larger penetration depth in mouse brain imaging compared with 1300 nm TD-OCM under the same sensitivity condition^18^. However, the penetration depth of the 1700-nm TD-OCM was limited to around 1 mm due to the relatively low signal sensitivity (93 dB). Recently, we developed 1700 nm spectral domain (SD) OCM with full range method to enhance the penetration depth, and the improved signal sensitivity (100 dB) enabled us to realize high-resolution deep tissue OCM imaging of pig thyroid glands with a penetration depth of 1.8 mm^19^.

As mentioned above, the previous studies of 1700 nm OCT/OCM have already reported that using the 1700 nm spectral band allows us to achieve a large penetration depth in turbid tissue imaging. In addition, our study also revealed that, even in deep tissue imaging, a high axial resolution of 3–5 µm was achieved by 1700 nm OCT using an SC source with a 300 nm spectral band (full width at half maximum (FWHM))^16^. However, although there is one report describing the image quality of a 1700 nm OCT system^10^, the image quality improvements that can be achieved when using the 1700 nm spectral band for OCM, for example, in terms of the signal-to-background ratio and the lateral resolution, still remain unclear. Note that, even in the 1700 nm OCT study, the imaging quality at a depth over 1 mm also has not been presented^10^. In OCM, the image quality is degraded presumably much more than in the case of OCT because imaging systems with higher NA objective lenses generally experience larger aberrations and light rays at larger angles are more likely to be scattered than those at smaller angles due to the longer travel length to the focus position^20,21^. Under the existence of the large aberration and multiple scattering effects, the laser focus becomes blurred and the diffraction-limited focus was no longer achieved. Besides, although OCM has greater confocal effects due to the use of high NA objective lenses than OCT, confocal gating effect becomes weaker under the existence of strong multiple scattering effects^22^. The reduction of the confocal gating effect degrades the lateral resolution of OCM and this degradation of the lateral resolution would be much more severe in OCM compared with OCT, which is based on imaging systems with low confocal gating effects. Although we have recently developed high-resolution TD and SD-OCM in the 1700 nm spectral band as mentioned above^18,19^, quantitative study of the imaging quality in deep tissue imaging (especially at a depth beyond 1 mm) has not performed yet.

In this paper, we quantitatively investigated the signal-to-background ratio (SBR) and lateral resolution in deep tissue OCM *en-face* imaging of brain samples with 1700 nm OCM, and compared the results with those of 1300 nm OCM. To perform this comparison at the same position in the samples and evaluate the image quality in brain imaging, we constructed a hybrid 1300 nm/1700 nm SD-OCM system with shared sample and reference arms. To show that the 1700 nm SD-OCM system has the deep tissue imaging capability like what we reported in ref.^18^, we first observed a fixed mouse brain and evaluated the signal attenuation coefficients. Then, we evaluated the imaging quality (the SBR and the lateral resolution) in deep tissue imaging by observing a reflective resolution target through a 0.5 to 1.5 mm-thick brain tissue phantom. In addition, high contrast deep tissue imaging of a mouse brain at a depth up to 1.8 mm was also demonstrated with high-resolution 1700 nm SD-OCM. To the best of our knowledge, as well as the study of the imaging quality, this is also the first report to observe small structures, such as neurons, in the such deep inside of mouse brain with high-resolution OCM.

## Results

**Figure 1:**
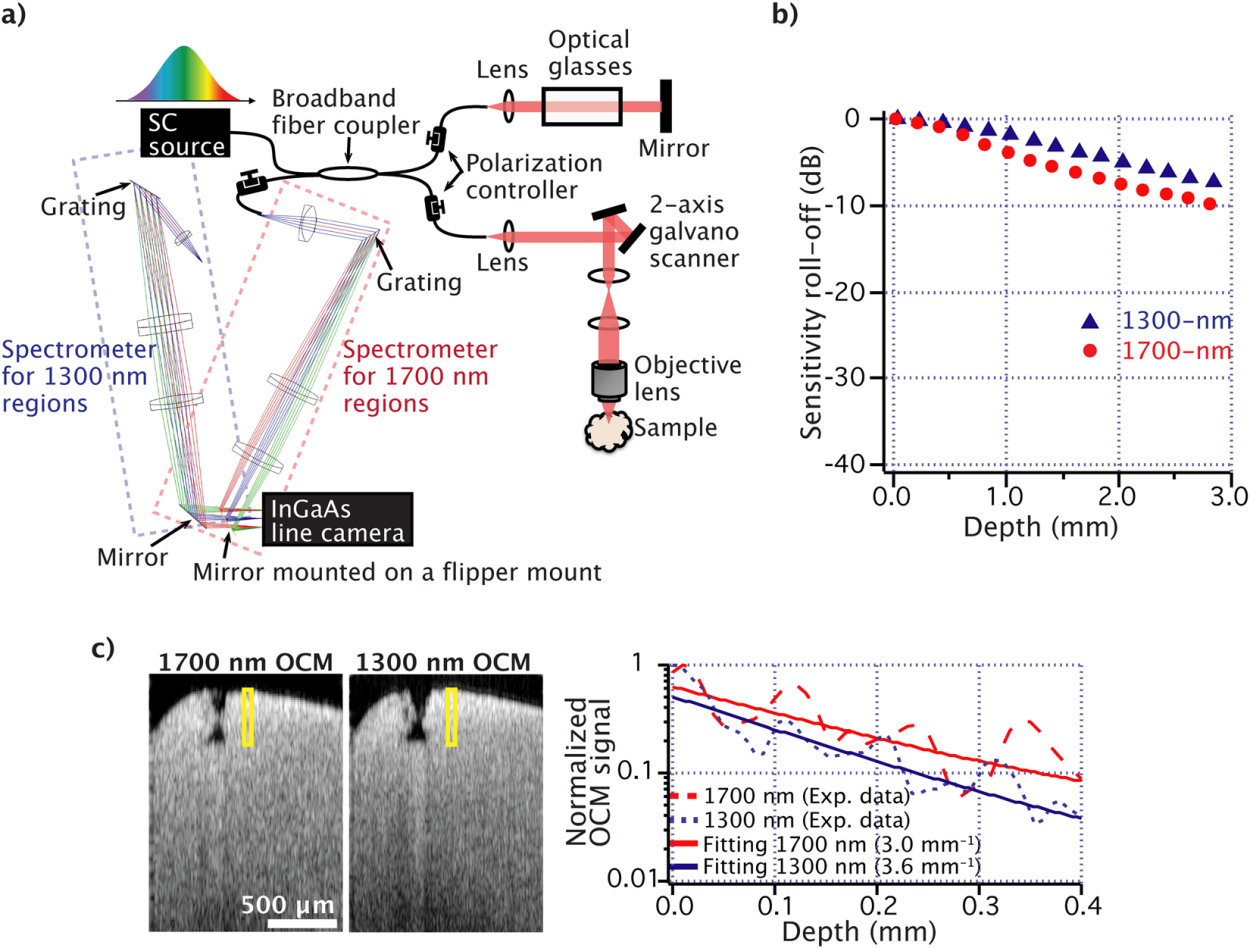
(**a**) Experimental setup for SD-OCM in the 1300 nm and 1700 nm spectral bands, (**b**) their sensitivity roll-off characteristics, and (**c**) cross-sectional images of a fixed mouse brain obtained with 1700 nm and 1300 nm OCM. The graph in (**c**) shows OCM signal intensities at different depths. These data were obtained from the yellow solid boxes in the brain images (averaged values along the horizontal direction in the yellow boxes). The dotted lines are measured data, and the solid lines are fitted curves.

Figure 1(a) shows the experimental setup for the hybrid 1300 nm/1700 nm SD-OCM system with shared sample and reference arms. As light sources, we used a polarized superluminescent diode (SLD) for 1300 nm OCM (S5FC1018P, Thorlabs) and our supercontinuum fiber laser source for 1700 nm OCM^13^. The laser beam was divided into the reference and sample arms by using a broadband fiber coupler. The sample arm consisted of an optical setup for laser scanning microscopy using a 2-axis galvanometer scanner. A 0.1 NA or 0.45 NA objective lens (LMPLN5XIR, LCPLN20XIR, Olympus) was used to focus the laser light on the samples and collect reflected and backscattered light from the samples. The reflected and backscattered light from the sample and reference arms interfered in the fiber coupler, and then the interference signal was measured with a spectrometer equipped with a high-speed InGaAs line scan camera (SU1024LDH-2.2RT-0250/LC, Goodrich). In our study, to achieve small sensitivity roll-off characteristics, we designed spectrometers with high spectral resolutions for both 1300 nm and 1700 nm OCM by using Zemax software. The spectral resolutions for 1300 nm and 1700 nm OCM were designed to be 0.05 and 0.09 nm, respectively. The spectrometer for 1300 nm OCM was composed of a 1210 lines/mm blazed diffraction grating (GR50–1210, Thorlabs) and two achromatic lenses (54–567, 45–419, Edmund Optics). The spectrometer for 1700 nm OCM was composed of a 940 lines/mm transmission diffraction grating (T-940CL, LightSmyth) and two achromatic lenses (45–419, 45–418, Edmund Optics). The detection wavelength ranges for 1300 nm and 1700 nm OCM were 1295–1345 nm and 1685–1755 nm, respectively. The line scan camera was shared by the spectrometers. In our system, the laser sources and spectrometers were switched manually by simply changing the fiber connections and flipping a mirror in front of the line scan camera. The chromatic dispersion and polarization mismatching between the sample and reference arms were compensated by using polarization controllers in both arms and optical glasses placed in the reference arm. By measuring interference signals from a silver-coated mirror, we confirmed that the sensitivity was 100 dB for both OCMs. Note that, after switching the spectral band, only slight or almost no adjustment of the polarization controllers was required to achieve 100 dB. Figure 1(b) shows the measured sensitivity roll-off characteristics for both OCMs. In this measurement, we detected a reflection light from a silver mirror and the signal intensities were measured by only changing the position of the reference mirror. During the measurement, the laser focus of the objective lens was fixed on the mirror surface. As shown in the result, we confirmed that the sensitivity roll-off was less than 4 dB/mm in our system.

Before we investigated the imaging quality in deep tissue imaging with 1700 nm OCM, we performed OCM imaging of a fixed mouse brain (from 12 week old mouse) to show the deep tissue imaging capability of the developed 1300 nm/1700 nm SD-OCM system. In this experiment, to avoid that the measurement is affected by sample movement during the spectral band switching and multiple scanning, we used a fixed mouse brain as a sample. This mouse brain was provided by Dr. Sumiko Kiryu-Seo (Grad. School of Medicine, Nagoya University, Japan). This mouse brain was surgically removed from a mouse under anesthesia and was fixed by perfusion. The mouse brain sample was prepared in accordance with the Animal Experimental Guides of Nagoya University Graduate School of Medicine. Figure 1(c) shows cross-sectional images of the fixed mouse brain, which were obtained with a 0.1 NA objective lens. In this measurement, after we confirmed the focus position was located on the sample surface by monitoring the signal intensity from the sample surface, the focus position was set at the outside near the sample surface by a mechanical sample stage equipped with a micrometer to avoid the saturation of the detector due to the strong reflection light from the sample surface. To reduce the influence of multiple-scattering effects, the attenuation coefficients were evaluated only in the first 400 µm region indicated by the yellow solid boxes in the brain images. The Rayleigh lengths, which were estimated from the lateral resolutions, were larger than 400 µm in both OCMs. To obtain the attenuation coefficients, we used curve fitting with an exponential decay model, *A*exp(−2 *µ*_t_*z*), where *µ*_t_ is the attenuation coefficient, and *z* is the depth position^6–8^. From the results, we confirmed that the attenuation coefficients of the 1300 nm and 1700 nm SD-OCM were 3.6 and 3.0 mm^−1^, respectively. The decrease of the OCM signals due to the sensitivity roll-off was corrected in both cases. This result indicated that this hybrid SD-OCM system allows us to compare the imaging quality under the conditions that the 1300 nm/1700 nm SD-OCM system has attenuation coefficients similar to those in our previous work and the literature^18,23^. Note that, although these measured attenuation coefficients in this work were slightly different from those in our previous work and the literature^18,23^, this is considered to be presumably due to the difference in the sample and experimental conditions.

**Figure 2:**
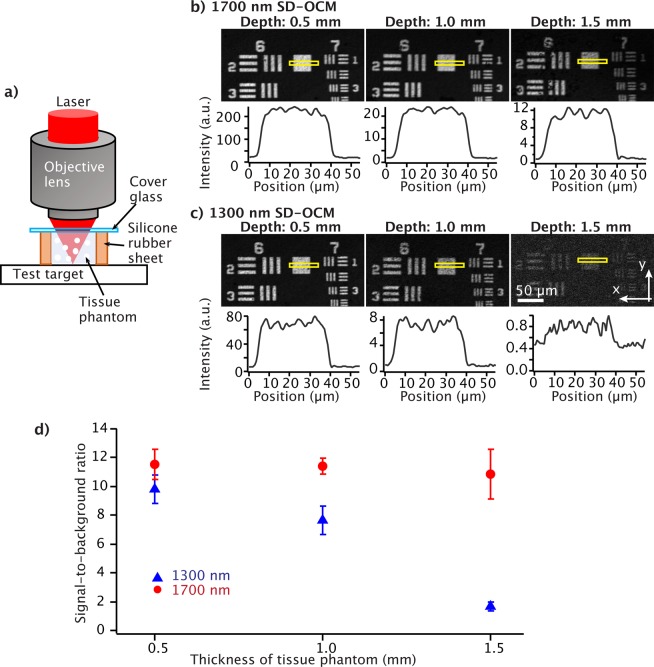
(**a**) Experimental configuration for the sample observation. OCM *en-face* images of the reflective resolution test target through 0.5, 1.0, and 1.5 mm thick tissue phantom obtained by (**b**) 1700 nm and (**c**) 1300 nm SD-COM. The images in (**b,c**) are shown in a linear scale. The graphs in (**b**) and (**c**) show the averaged intensity profiles of areas indicated by the yellow boxes in the OCM *en-face* images. (**d**) The relationship between SBR and thickness of the tissue phantom.

To evaluate the SBR and the lateral resolution in OCM *en-face* imaging, we observed a reflective resolution test target (R3L3S1P, Thorlabs) through 0.5, 1.0, and 1.5 mm thick tissue phantoms of mouse brain cortexes. A 0.45 NA objective lens was used for this experiment. The focus position was set on the surface of the test target by finding the depth where the intensity of the reflection light from the test target becomes highest. In this experiment, we used a 1 µm polystyrene bead solution (concentration: 5.4 × 10^9^ ml^−1^), which is reported to have the effective attenuation length similar to that of mouse brain cortex^24,25^. The thickness of the polystyrene bead solution layer was set to 0.5, 1.0, or 1.5 mm by using silicone rubber sheets. The polystyrene bead solution was placed on the reflective resolution test target as shown in Fig. 2(a). Although the observation position was slightly changed when we replaced the silicone rubber sheet to change the thickness of the tissue phantom, the effect of the observation position difference can be ignored because this tissue phantom was a homogeneous mixture of polystyrene beads and water. This is the reason why we choose this sample for this quantitative study. Figure 2(b,c) show OCM *en-face* images and their intensity profiles in areas indicated by the yellow dotted boxes in the images. From the intensity profiles, we calculated the SBRs and confirmed that the SBR of 1700 nm OCM remained almost unchanged, whereas that of 1300 nm OCM dramatically decreased as the thickness of the tissue phantom increased (Fig. 2(d)). In this experiment, although we used the 1300 nm SD-OCM with the sensitivity of 100 dB, it is reported elsewhere that it is possible to achieve slightly higher sensitivity than 100 dB in 1300 nm OCM^4^. The SBR would be improved if the sensitivity becomes higher, while the degradation behavior of the SBR would be similar to that in this result. Therefore, 1700 nm OCM still provides higher SBR in deep tissue imaging if the sensitivity of 1300 nm OCM is only a few dB higher than that of 1700 nm OCM.

**Figure 3:**
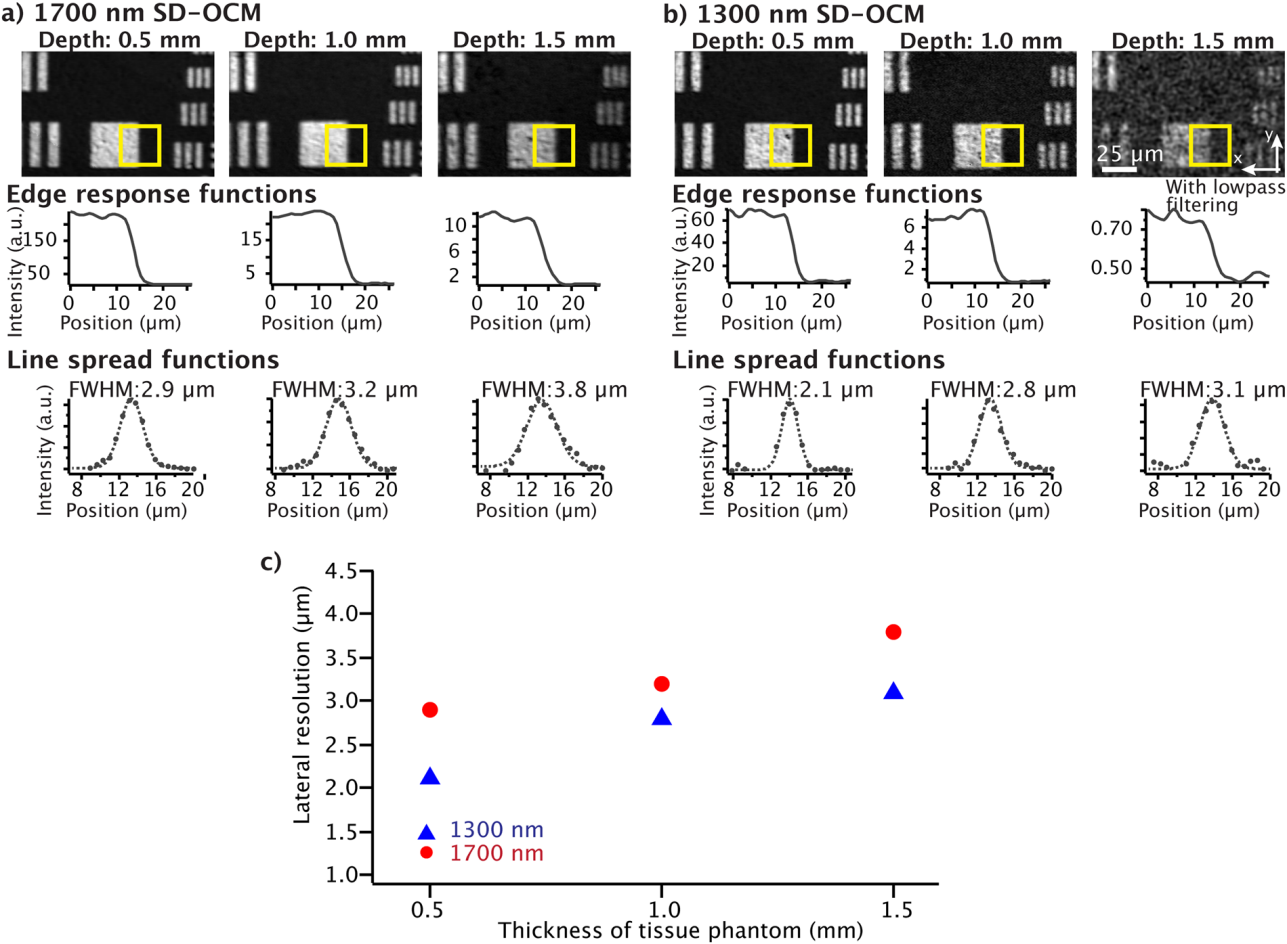
OCM *en-face* images obtained by (**a**) 1700 nm and (**b**) 1300 nm SD-OCM. The graphs in (**a**) and (**b**) show the averaged intensity profiles (edge response functions) of the areas indicated by the yellow boxes (above) and their corresponding line spread functions (bottom). The images in (**a,b**) are shown in a linear scale. The dotted lines in the line spread functions show their gaussian fits. (**c**) The relationship between the lateral resolution and thickness of the tissue phantom.

We then evaluated the lateral resolution by observing line spread functions in the reflective resolution test target (Fig. 3). From the OCM *en-face* images, we obtained the averaged intensity profiles (edge response functions) of the  areas indicated by the yellow boxes in the images. Then, we calculated the corresponding line spread functions from the averaged intensity profiles (Fig. 3a,b). In this experiment, for 1300 nm OCM *en-face* imaging with a 1.5 mm-thick tissue phantom, we applied lowpass filtering to reduce noise because a sufficiently high signal-to-noise ratio was not achieved to calculate the line spread function. The cutoff spatial frequency for the lowpass filtering was determined by the theoretical lateral resolution so that the lowpass filtering did not degrade the lateral resolution. As shown in Fig. 3c, the lateral resolution of both SD-OCM was gradually degraded with the increase of the thickness of the tissue phantom and the degradation percentages of the lateral resolution for 1700 nm and 1300 nm SD-OCM were 31% and 47%, respectively. From this result, although the use of 1700 nm spectral band slightly moderated the degradation of the lateral resolution, we confirmed that there is no significant difference compared with the result in the SBR experiment.

Although high lateral resolution was achieved in both OCMs, as shown in Fig. 3, our results revealed that the use of the 1700 nm spectral band for OCM provided a significantly improved SBR in deep tissue imaging, compared with the 1300 nm spectral band. This is considered to be mainly due to the lower scattering efficiency in the 1700 nm spectral band than that in the 1300 nm spectral band. In the signal detection from deep parts of turbid scattering tissues, there is loss of the reflected and backscattered light due to multiple light scattering in the samples, which reduces the amount of light collected by an objective lens^22^. In addition, light scattering in the samples also reduces the coupling efficiency of the reflected and backscattered light into the optical fiber of the fiber coupler because it causes distortion of the focus spot at the core of the optical fiber, which works as a confocal pinhole. Therefore, it is considered that using light with a lower scattering coefficient contributes to improvement of the SBR in OCM imaging. The degradation behavior of the SBR and the lateral resolution might be different in observations of actual tissue samples, which usually have more complicated structures than the tissue phantom. However, to some degree, the results are considered to represent the behavior of SBR and lateral resolution in practical situations, because the effective attenuation length of the tissue phantom is similar to that of mouse brain cortexes.

Finally, we demonstrated high-contrast deep tissue imaging of a mouse brain. For this experiment, we used a full-range high-resolution 1700 nm SD-OCM system using an SC fiber laser source with a 300 nm spectral band (full width at half maximum)^19^. For this OCM system, we used another custom-built spectrometer consisting of a 150 lines/mm blazed diffraction grating (015–200, Shimadzu) and two achromatic lenses (AC508-040-C and AC508-050-C, Thorlabs)^14^. The line scan camera was the same as that in Fig. 1. In this OCM system with a 0.45 NA objective lens, the lateral and axial resolutions were 3.4 µm and 3.8 µm in tissue, respectively. To the best of our knowledge, the detection volume is the smallest reported among Fourier-domain optical coherence imaging techniques in the 1700 nm spectral band. The lateral resolution was slightly lower than that shown in Fig. 3. This is presumably due to the larger chromatic aberration effect caused by using the SC light with the wider spectral band than that used for experiments in Fig. 3. The sensitivity of this system was 100 dB. Although the experimental setup of this OCM system was almost the same as that in Fig. 1, this SD-OCM system had a large sensitivity roll-off (−20 dB/mm) because it sacrifices spectral resolution to achieve high axial resolution. To access deep parts in tissue with this high-resolution 1700 nm SD-OCM, we employed a full-range method, which enabled us to suppress the formation of a coherence ghost image and set the zero-delay position inside the sample. Details of the full-range method are given in ref.^14^.

**Figure 4:**
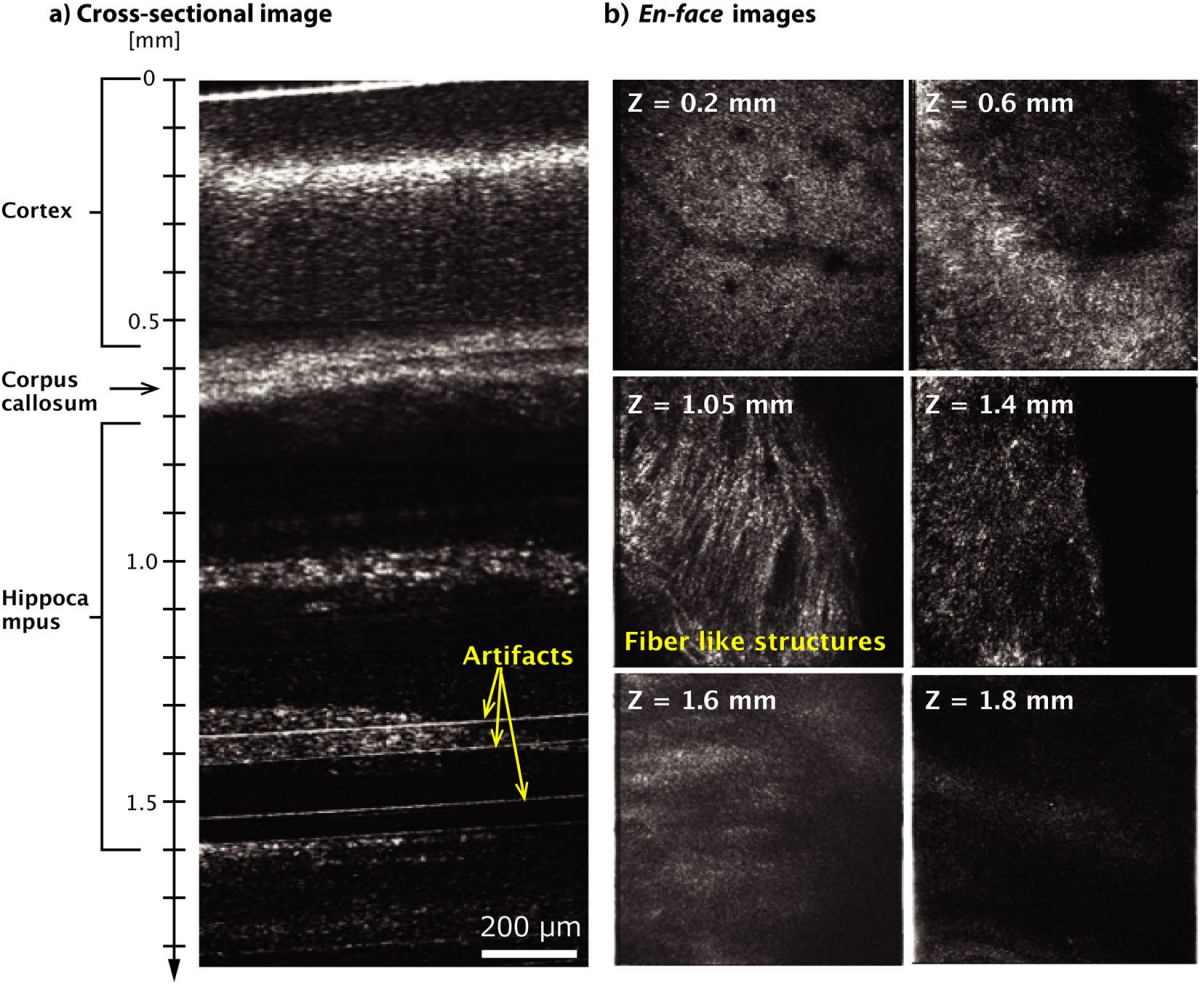
(**a**) Cross-sectional images and (**b**) *en-face* images of a mouse brain obtained with full-range high-resolution 1700 nm SD-OCM.

Figure 4a,b show the cross-sectional image and *en-face* images of a mouse brain (12 weeks) at different depths. The mouse brain was purchased from a company (Japan Lamb Co. Ltd.) and it was delivered in cold storage (4 °C) shortly after surgical removal from the mouse. Because it is reported that fixation process of tissue samples increases scattering coefficients by protein cross linkage, sample dehydration, and shrinkage^26^, we used an unfixed sample in this experiment to demonstrate mouse brain imaging with 1700 nm OCM under the condition more similar to that in practical tissue imaging. During OCM imaging, the mouse brain was immersed in phosphate buffered saline solution to prevent the sample from drying out. In this experiment, we obtained three-dimensional (3D) data sets at five different imaging depths and combined them to construct a single 3D volumetric data set. In this experiment, the observation area in each image at the different imaging depths was slightly overlapped like the case in ref.^14^ and then determined the connecting depth from the images. From the constructed 3D volumetric data set, we obtained the cross-sectional and *en-face* images. As shown in Fig. 4a, thanks to the full-range method, a penetration depth of 1.8 mm was achieved. In the *en-face* image at a depth of 1.05 mm, fiber-like structures were observed (Fig. 4b). The fiber-like structures are presumably highly myelinated axons in white matter, which are known to be strong light scatterers because they have lipid-rich myelin sheaths^27^. Similar myelin fiber-like structures were also reported in refs^4,18^. In addition to the large penetration depth, although the degradation of the spatial resolution was caused by sample-induced optical aberrations and chromatic dispersion mismatching, we confirmed that the structures of the mouse brain were visualized with sufficiently high SBR. These results indicated that the large penetration and high SBR imaging capabilities of 1700 nm OCM are considerably useful for biomedical studies requiring the observation of turbid scattering tissues.

## Conclusion

In this paper, we have reported a quantitative study of the image quality in 1700 nm optical coherence microscopy (OCM), in terms of the signal-to-background ratio (SBR) and the lateral resolution. By using a hybrid 1300 nm/1700 nm spectral domain (SD) OCM system with shared sample and reference arms, we evaluated the image quality at the same position in samples. Using tissue phantoms and a resolution test target, our results showed that using the 1700 nm spectral band for OCM in deep tissue imaging offered an SBR around 6-times higher than the 1300 nm spectral band, although there is no significant difference in the degradation behavior of the lateral resolutions of both OCMs. We also demonstrated deep tissue imaging of a mouse brain (unfixed) at a depth up to 1.8 mm with sufficiently high SBR by using high-resolution 1700 nm SD-OCM. The imaging capabilities of this system, with large penetration depth and high SBR, would also be beneficial for image analysis based on signal processing, such as optical elastography^28^, which requires images with sufficiently high SBR. The SBR and the lateral resolution could be further improved by adopting adaptive optics^20^ and numerical approaches to compensate for chromatic dispersion caused by the sample^16,29^.
